# A clinical-pathological and survival study of oral squamous cell carcinomas from a population of the north of Portugal

**DOI:** 10.4317/medoral.19090

**Published:** 2013-10-13

**Authors:** Luís S. Monteiro, José B. do Amaral, José R. Vizcaíno, Carlos A. Lopes, Fernando O. Torres

**Affiliations:** 1 PhD, MSc, DDS. Medicine and Oral Surgery Department, Dental Sciences Group – Health Sciences Research Centre, Instituto Superior de Ciências da Saúde Norte, CESPU, Paredes, Portugal; 2 PhD, MD. Stomatology Department, Centro Hospitalar do Porto, Hospital de Santo António-Porto, Portugal; 3 MD. Pathology Department, Centro Hospitalar do Porto, Hospital de Santo António-Porto, Portugal; 4 PhD, MD. Molecular Pathology and Immunology Department, Instituto de Ciências Biomédicas Abel Salazar (ICBAS), Porto University, Porto, Portugal; 5 PhD, MD. Pathology Department, Instituto Superior de Ciências da Saúde Norte, CESPU, Paredes, Portugal

## Abstract

Objectives: Our aim was to analyze the clinical, pathological, and outcome characteristics of oral squamous cell carcinomas (OSCC) from a population of the north of Portugal. Material and Methods: We conducted a descriptive study of 128 OSCC diagnosed between the years of 2000 and 2010 in the Centro Hospitalar do Porto. Through of the review of the clinical records we studied several clinical, pathological, and outcome variables. The overall survival (OS) and disease-free survival (DFS) were analyzed by Kaplan-Meier method and log-rank test. Cox regression method was used for multivariate analysis. Results: Of 128 patients with OSCC, 83 (64.8%) were male and 45 (35.2%) were female, (mean age of 62.13±15.57 years). The most affected location was the tongue (n=52; 40.6%). The most common cause of reference was a non-healing ulcer (n=35; 28.9%) followed by oral pain (n=27; 22.3%). Sixty (60.6%) patients were tobacco consumers and 55 (57.3%) alcohol consumers. The cumulative 3-years OS rate was 58.6% and DFS was 55.4%. In multivariable analysis for OS, we found an adverse independent prognostic value for advanced tumour size (p<0.001) and for the presence of perineural permeation (p=0.012). For DFS, advanced stage tumours presented adverse independent prognostic value (p<0.001). Conclusions: OSCC occurred most frequently in males, in older patients, and in patients with tobacco and/or alcohol habits. TNM and tumour stage additionally to the perineural permeation were the most important prognostic factors for the survival of these patients, contributing to identify high-risk subgroups and to guide therapy.

** Key words:**Squamous cell carcinoma, mouth neoplasms, oral cancer, oral pathology, prognosis.

## Introduction

Oral cancer is a major public health problem worldwide. Oral and pharyngeal grouped together are the sixth most common cancers in the world (1). An estimated 263,900 new cases and 128,000 deaths from lip and oral cavity cancer occurred in the year of 2008 worldwide (2). In Portugal, a total of 1200 cases of lip, oral and oropharynx cancers were reported in 2007, 949 (78.61%) in males and 251 (21.4%) in females. Moreover, in the last decade there has been an increasing trend for oral cancer in Portuguese population in both sexes and especially in the female group (3).

Almost 90% of oral cancers are squamous cell carcinomas (3). Smoking, alcohol use, and HPV infections are the major risk factors, with an attributable risk of oral cancer due to both tobacco and alcohol of 80% (4).

Despite recent advances in the detection and treatment of cancer, visual accessibility of the oral mucosa and the scientific knowledge on cancer risk factors, oral cancer carried a low survival rate (near 50%) in the last few decades (1).

Treatment consists mainly in surgery, radiotherapy and/or chemotherapy (5). However these treatment modalities are often associated with collateral effects that diminish considerably the quality of life of the patients (6). Therefore is important to identify and stratify patients with greater precision to the most appropriate choice of a treatment plan, avoiding excessive treatment in patients with low risk of recurrence and excessively conservative treatments in patients in whom the risk of recurrence is high.

The purpose of this study is to examine the clinical and pathological characteristics of oral squamous cell carcinomas (OSCC) and analyze their influence in the outcome of these patients.

## Material and Methods

-Patients population

This was a retrospective study of 128 consecutive patients diagnosed and treated for primary OSCC at the *Centro Hospitalar do Porto – Hospital de Santo António, Porto, Portugal*, between 2000 and 2010. The study was approved and performed according to the institutional review board of the hospital. We include all consecutive primary OSCC located in the lip mucosa or oral cavity (C00.3-C00.5, C01-06). Patients were excluded when lacking relevant clinical and follow-up information or without histological confirmation of their diagnosis. From patient’s records we obtained patient’s age, gender, tumour location, first clinical manifestation of the disease, clinical tumour presentation, history of potentially malignant disorders, tobacco and alcohol habits, tumour stage, primary treatment, tumour grade, surgical margin status, vascular, lymphatic and perineural invasion, and follow-up information.

The tobacco and alcohol habits were categorized into the groups: consumers, non consumers and ex-consumers. Patients who smoked cigarettes (or equivalents) at the rate of 20 or more per day were considered heavy smokers (7). Those who drank alcohol at 30.0 g/day or more (for men) or 15.0 g/day or more (for women) were considered as heavy drinkers.

Tumour stage was reclassified according to the 7th edition of the classification of malignant tumours of American Joint Committee on Cancer (8).

Treatment options were registered and grouped for statistical analysis into the categories: 1 – surgery alone; 2 – surgery and adjunctive radiotherapy (external-beam radiotherapy, 55-66Gy); 3 – chemotherapy (5-fluorouracil and cisplatin) followed by surgery; 4 - chemotherapy followed by surgery and adjunctive radiotherapy; 5 – other treatments including radiotherapy alone, chemotherapy alone or chemoradiotherapy; and 6 – support treatment.

Hematoxylin-eosin-stained slides were available for all tumours to confirm the initial diagnosis. Tumour grade was reclassified into well differentiated (G1), moderately differentiated (G2), and poorly differentiated (G3) OSCC. Inspection for possible presence of vascular (venous), lymphatic and perineural invasion reported as present or absent, was performed on each sample. Surgical margin were classified according Sutton et al. (9).

-Statistical analysis

The descriptive results were reported as absolute and relative frequencies. Associations between categorical variables were analysed using Chi-square test. Overall survival (OS) was defined as the time interval (months) between primary treatment and death by oral cancer or last follow-up. Disease-free survival (DFS) was defined as the time interval (months) between primary treatment and the first recurrence (whether local, regional or distant). The Kaplan-Meier method was used to plot survival curves and their prognostic effect was tested using the log-rank test. Variables with significant effects in the univariate analyses were entered into Cox proportional hazards model to investigate the independent effects of these variables.

Differences were considered statistically significant at p<0.05. Statistical analyses were carried out using IBM SPSS Statistics version 21.0 software (IBM Corporation, NY, US).

## Results

-Clinical and pathological findings

The study group included 128 patients, 83 men (64.8%) and 45 women (35.2%), with a ratio male:female of 1.8:1, whose ages ranged from 21 to 96 years, with a mean age of 62.13 ±15.57 years. There were 17 (13.3%) cases below the 45 years of age. Men were younger than women. Fifty men (60.2%) were under 62 years and 32 (71.1%) women had more than 62 years old (p=0.001).

The most common clinical manifestation that brought the patient to the hospital was a non-healing ulcer in 35 cases (31.8%) followed by oral pain (n=27; 24.5%), odynophagia (n=12; 10.9%), mass or nodule (n=11; 10%), persistent white or red patch (n=7; 6.4%), dysphagia (n=6; 5.5%), cervical mass (n=5; 4.5%), prosthesis that fails to fit (n=3; 3.6%), oral haemorrhage (n=2; 1.8%), trismus (n=1; 0.9%%), and foreign body sensation in one patient (0.9%). This information was unknown in 18 cases.

The most frequently affected location was the tongue (n=52; 40.6%) followed by the floor of the mouth (n=19; 14.8%), gingiva (n=15; 11.7%), hard palate (n=10; 7.8%), retromolar region (n=13; 10.2%), labial mucosa (n=11; 8.6%), and buccal mucosa (n=8; 6.3%).

In the cases with tobacco information (n=99) we found the report of tobacco consumption in 60 (60.6%) patients, of which 58 (58.6%) were heavy smokers. Seven (7.1%) patients were tobacco ex-consumers and 32 (32.3%) were non consumers. Alcohol consumption was registered in 55 (57.3%) patients of whom 43 (44.8%) were heavy drinkers. Forty (41.7%) patients were alcohol non consumers and one (1%) patient was ex-consumer. In 32 cases alcohol information was not available. Forty-two (43.8%) patients had simultaneous tobacco and alcohol consumption of which 36 (37.5%) were both heavy smokers and heavy drinkers.

The relation between tobacco and alcohol habits with age, gender and tumour location is shown in  Table 1. We found a higher prevalence of smokers in males (p<0.001), and in the youngest age group (p<0.001). Moreover, males showed predominant alco-hol misuse habits comparing to females (p<0.001). More than a half of the patients with tumours located on the floor of the mouth, retromolar trigone, and tongue were tobacco consumers ( Table 1).

**Table 1:**
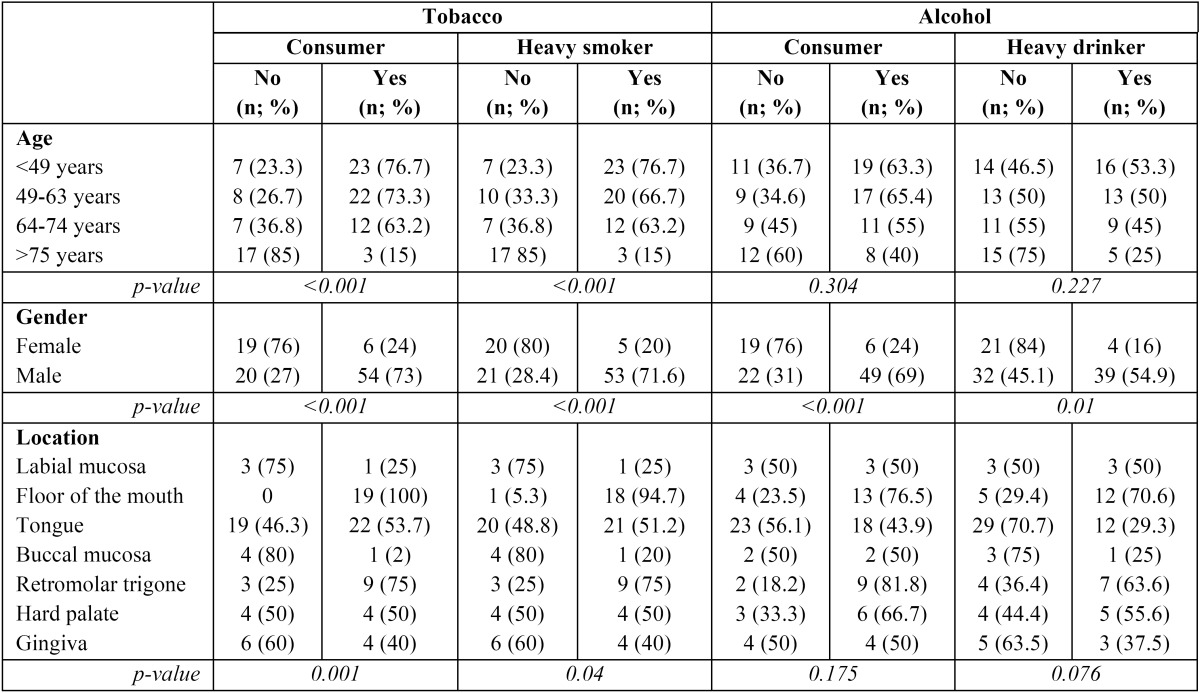
Associations between tobacco/alcohol habits with age, gender and tumour location.

On the first intra-oral clinical examination, the tumour had an ulcer presentation in 55 cases (46.6%), an exophytic (verruciform) appearance in 19 (16.1%) cases, an ulcer-exophytic appearance in 37 (31.4%) cases and a patch (white or red) presentation in 7 (5.9%) cases in the 118 cases with this information. History of leukoplakia was recorded in 17 (13.3%) cases, erythroplakia in 3 (2.3%) and actinic cheilitis (on lip) in 3 (2.3%) cases.

Tumours were T1 in 34 cases (26.6%), T2 in 47 cases (36.7%), T3 in 10 cases (7.8%), T4a in 31 cases (24.2%), and T4b in 6 cases (4.7%). Fifty patients (39%) had tumour nodal involvement. N1 status was found in 19 (14.8%) patients, N2 in 26 patients (20.3%), and N3 in 5 patients (3.9%). One subject (0.8%) had a distant metastasis (lung) at time of diagnosis.

In the view of this, 26 patients (20.3%) presented stage-I tumours, 32 patients (25%) had stage-II tumours, 20 patients (15.6%) presented stage-III tumours, 40 patients (31.3%) had stage-IVA tumours, 9 (7%) stage-IVB tumours, and one (0.8%) had a stage-IVC tumour.

Fifty-eight patients (45.3%) were treated with surgery alone, 37 (28.9%) with surgery and adjunctive radiotherapy, 3 (2.3%) with chemotherapy followed by surgery, 7 (5.5%) with chemotherapy followed by surgery and adjunctive radiotherapy, and 10 (7.8%) with other treatments including radiotherapy alone, chemotherapy alone or chemoradiotherapy. Thirteen patients (10.2%) received only support treatment.

Tumours were graded as well-differentiated in 74 (57.8%) cases, moderately differentiated in 45 (35.2%), and poorly differentiated in 9 (7%) cases. Of the 105 patients submitted to surgery, there were 60 cases with report of surgical margin status. Eleven (18.3%) patients had tumour cells in the surgical margins, 33 (55%) were free of tumour cells, and 16 (26.7%) were close to tumour cells. We found vascular invasion in 11 (11.2%) cases, perineural permeation in 14 (14.3%) cases, and lymphatic invasion in 19 (19.4%) in the 98 cases wherethis analysis was available.

-Clinical Outcome

**Figure 1:**
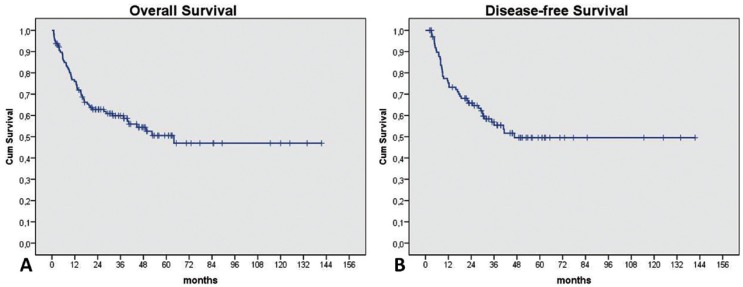
Kaplan-Meier plots to overall survival (A) and disease-free survival (B).

At the end of our study, 68 patients (53.1%) were alive without oral cancer, 2 patients (1.6%) were alive with oral cancer, 56 (43.8%) had died as a result of the oral cancer and 2 patients (1.6%) had died as a result of other non-cancer disease. The follow-up mean for all patients was 33.2±28.8 months. The cumulative 3-years OS rate was 58.6% and DFS was 55.4%. At 5-years OS and DFS rates corresponded to 50.5% and 49.5%, respectively (Fig. 1).

**Figure 2:**
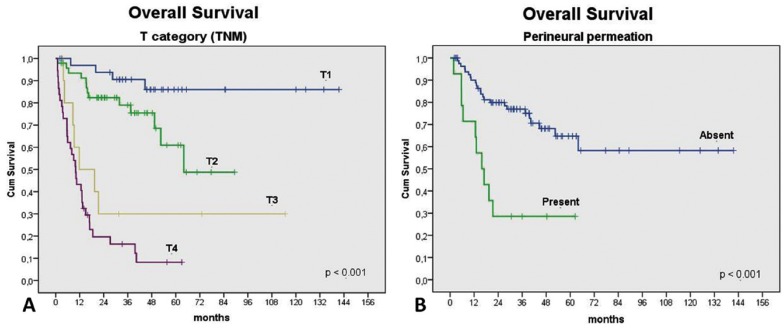
Kaplan-Meier plots to overall survival according T category of TNM classification (A) and perineural permeation (B).

**Figure 3:**
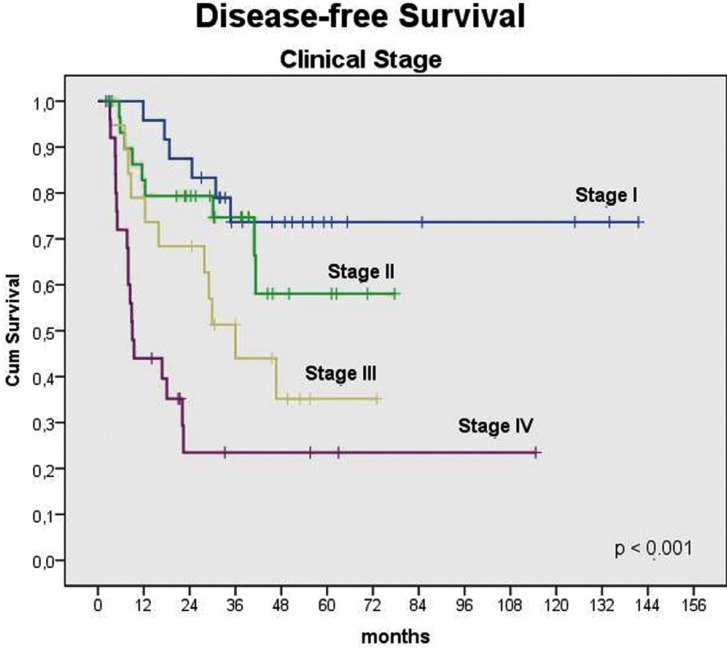
Kaplan-Meier plots to disease-free survival according clinical tumour stage.

In univariable analysis, tumour location (p=0.034), T status (p<0.001) (Fig. 2), N status (p<0.001), clinical stage (p<0.001), surgical margins (p=0.001), treatment option (p<0.001), histological grade (p<0.001), vascular invasion (p=0.005), and perineural permeation (p<0.001) (Fig. 2) were statistically correlated to OS. The same was observed with reference to DFS for T status (p=0.001), N status (p<0.001), clinical stage (p<0.001) (Fig. 3), treatment option (p=0.01), surgical margins (p=0.038), histological grade (p=0.041), and perineural permeation (p=0.007) ( Table 2).

**Table 2:**
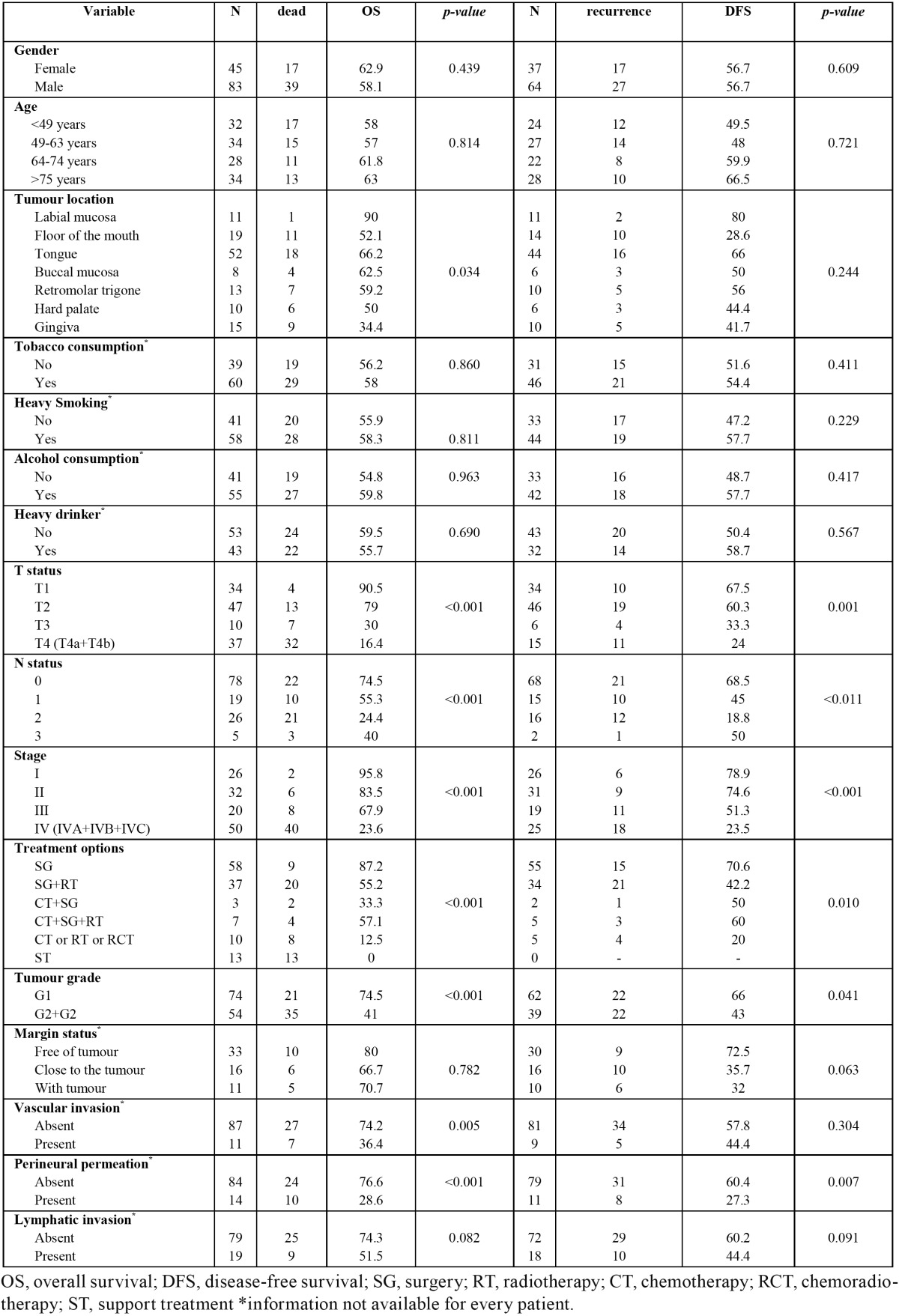
Kaplan-Meier estimates of probability of overall survival and disease-free survival at 3 years of follow-up (univariate analysis).

In multivariable analysis for OS, we found an adverse independent prognostic value for advanced tumour size (T) and for the presence of perineural permeation ( Table 3). For DFS, advanced clinical stage presented adverse independent prognostic value ( Table 4).

**Table 3:**
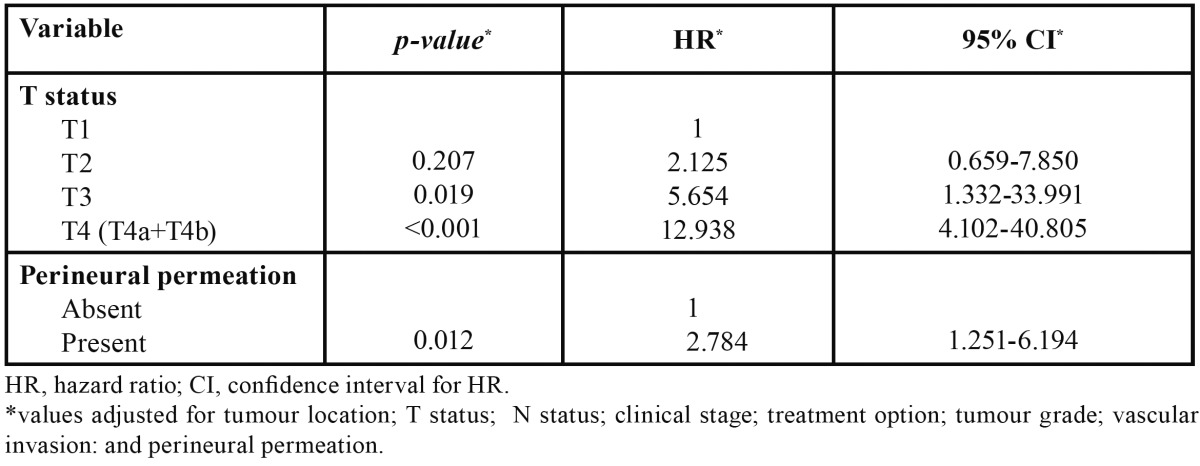
Variables with significant independent effect in the multivariate Cox regression analysis of the overall survival.

**Table 4:**
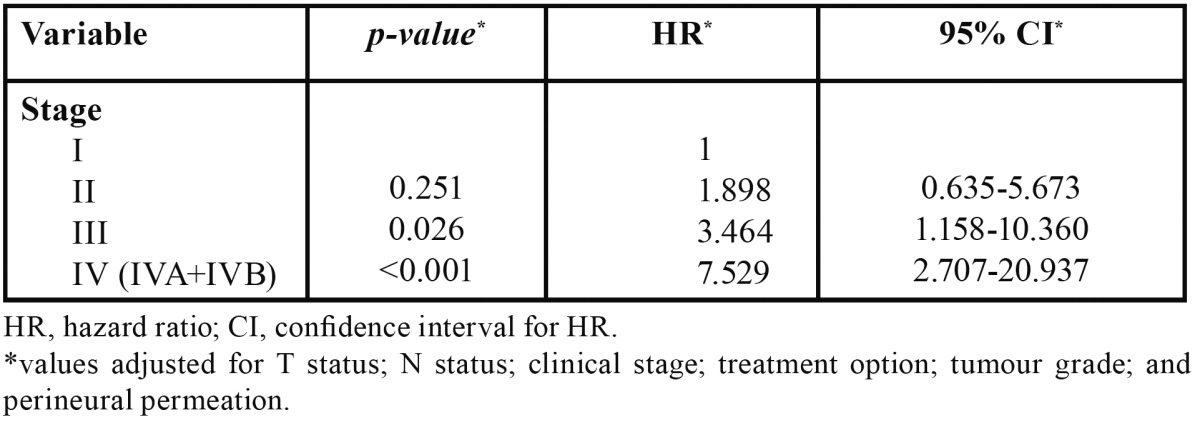
Variables with significant independent effect in the multivariate Cox regression analysis of the disease-free survival.

## Discussion

Few studies have characterised oral cancers in the Portuguese population (10,11). With this in mind, we analysed the clinical and pathological features and the outcome of a series of patients with OSCC from a central hospital in the north of Portugal.

We observed that oral cancer was twice more frequent in males than females (ratio 1.8:1) which is in accordance with the predominant male frequency of this cancer reported in literature (12). However this ratio was lower than the reported by others (10,11,13,14). This could be attributed to the increasing trend of oral cancer in women in Portugal or simply be a result of sample constitution (3). The mean age (62 years) found in the present study is compatible with the majority of the published studies (10,12,15). There were 13.3% of the patients with less than 45 year-old, result that has been described with increasing frequency in the literature (16-18).

Tobacco and/or alcohol habits were present in more than a half of the patients and were predominant in males. This is an expected result in squamous cell carcinomas of the oral cavity, reflecting the importance of these habits as risk factors in oral cancer (4,10). Therefore, primary prevention measures must be strengthened to a decrease of such habits.

In the last decades, an important role of HPV on the aetiology of oral cancer has been suggested (4). However, in many cases HPV detection in oral tumours specimens is not routinely done probably due to the high cost of HPV DNA identification techniques. Some authors proposed p16 immunohistochemical detection as a marker of HPV infection (19). We think that this could be a more cost-effective option as a routine first-line marker of HPV in oral cancers specimens. It is our objective, in the future, to analyse HPV status with p16 immunostaining and with HPV in situ hybridization techniques in these tumours.

More than a half of the patients had an advanced tumour stage at presentation. This is in accordance with the results from other Portuguese studies (10) but contrast with some recent publications regarding oral cancer in Spain where most of patients had stage I or II tumours (13,20). One of the reasons for this may be related with the possible low awareness of the population for oral cancer (7).

The rates of OS and DFS at 60 months were 50.5% and 49.5%, respectively, which are in accordance with the described survival rate for oral cancer in the most parts of the world (1,12,21-23). However, recent US data showed a statistically significant improvement in overall survival among patients treated for OSCC from 54% in 1987-1989 to 63% in the 2001-2007 timeframe (24).

Age and gender did not influence the OS and DFS, similar to the observed in several studies (25-27). Tobacco and alcohol consumption also did not influence the outcome of these patients, as observed by Vallecillo-Capilla et al. (13).

Tumour staging, using TNM classification, is the most common and accepted prognostic system for patients with cancer (28). Several studies have demonstrated the independent prognostic value of TNM classification on oral cancer (13-15,29). We confirmed this in the present work. The T and N categories and the tumour stage showed a highly significant association with OS and DFS in the univariate analysis. In the multivariate analysis T category had an independent prognostic value on the OS. Patients with T4 tumours showed a hazard ratio value 13 times higher than patients with T1 tumours. Tumour stage revealed an independent prognostic value in disease-free survival. Patients with stage IV tumours showed a hazard ratio value 8 times higher than patients with stage I tumours. Moreover as we already saw in this study, most of the patients were diagnosed in advanced stages already. This reflects the need for early diagnosis of this pathology at least in the north of Portugal. This is a concerning problem that needs to be strengthened in Portugal with several measures such as awareness and knowledge campaigns on oral cancer, and oral cancer screenings.

The histologic grade showed, in the present work, that well differentiated tumours had a better outcome on univariate analysis than less differentiated tumours. However, this variable lost significance in multivariate analysis. Nevertheless, Kademani et al (27) reported the independent value of the degree of cellular differentiation as a predictor of the survival of patients with OSCC.

The presence of perineural permeation was a strong indicator of an adverse OS with an independent effect on multivariate analysis. The perineural spread is reported at a frequency between 6 and 30% in several head and neck carcinomas and regarded as an important prognosis factor (30).

In conclusion, this study indicates that OSCC occurred most frequently in males, in older patients, and in patients with tobacco and/or alcohol habits. Non-healing ulcers and oral pain were the most common reasons for the first clinical examination, very often in an advanced tumour stage. TNM and tumour stage classification and perineural permeation were the most important prognostic factors for the survival of these patients, contributing to identify high-risk subgroups and to guide therapy.
